# Eyebrows

**Published:** 1881-10

**Authors:** 


### EYEBROWS.

"ITEM—two eyebrows, indifferent, badly put on," concluded a young lady, talking with true feminine charity of a fair sister, then going through the mazes of a lanciers on the middle of the floor.

"What do you mean?" said the reporter.

"Why, they are a misfit."

A long look, a steady look and a look altogether at the eyebrows in question did indeed indicate a something wrong about them, but what it was was not so evident.

"Did you really mean that they are false?"

"I did."

"And that the resources of civilization have put within the reach of lovely woman the means of circumventing nature when the good dame is parsimonious in the matter of eyebrows?"

"Yes, that is what I mean."

"But how are they put on, and how are they kept on?"

"You never would understand it if you did not see how it was done; the process is called re-enforcing the eyebrows. You know most girls have eyebrows, as far as they go, but—especially in blondes—this feature is defective; while even brunettes would generally wish to have more eyebrows than they can grow naturally."

"Why?"

"Because it sets off the eye, and takes off that appearance of boldness or bulginess which an unbrowed eye always has. If you ever see a young lady at her toilet—which, of course, you haven't—you will notice that the very last thing she does is to light a match and burn it so as to leave as much charcoal as possible. This little crayon is then wet with—with the dew on her rosebud lips, and drawn lightly and skilfully across her brows in the true arch of Juno. The black deposit is then rubbed in beneath the tiny hairs, having only the effect of darkness, and the result is enough to make the eyebrows stand out to the proper relative place in the precision of the features.

"I get that effect with charcoal; Jennie gets it with more hair. When they don't fit there is a look about them which is very unnatural, and by which they can be detected at once."

"But how are they put on?"

"It is a very complicated process and an elaborate one. The eyebrows must be parted down the centre, and this false bit, which is just an edge of hair carefully bandolined along the bottom, stuck in and combed lengthways with the eyebrows. It is extremely difficult to do this properly, taking a great deal of time and trouble, and then when it is done the whole business may be brushed out by accident. A girl has to take a whole lot of chances anyhow if she wants to look pretty."—*St. Louis Post-Dispatch.*

